# Screening of *Jiangshui*-originated lactic acid bacteria with high hypoglycemic properties and study on functional properties of Jerusalem artichoke *Jiangshui*

**DOI:** 10.3389/fmicb.2025.1660938

**Published:** 2025-09-16

**Authors:** Yan-Wen Gui, Qing-kai Jin, Hui Yao, Mohamed S. Sheteiwy, Afrah E. Mohammed, Modhi O. Alotaibi, Si-Jing Chang, Xian-Gang Meng

**Affiliations:** ^1^Department of Bioengineering, School of Biological and Pharmaceutical Engineering, Lanzhou Jiaotong University, Lanzhou, China; ^2^Department of Integrative Agriculture, College of Agriculture and Veterinary Medicine, United Arab Emirates University, Abu Dhabi, United Arab Emirates; ^3^Department of Biology, College of Science, Princess Nourah bint Abdulrahman University, Riyadh, Saudi Arabia; ^4^Microbiology and Immunology Unit, Natural and Health Sciences Research Center, Princess Nourah bint Abdulrahman University, Riyadh, Saudi Arabia

**Keywords:** lactic acid bacteria, aroma-producing yeast, fermentation, hypoglycemic, Jerusalem artichoke *Jiangshui*

## Abstract

The current study was designed to evaluate and characterize lactic acid bacteria (LAB) with high hypoglycemic properties isolated from Northwest *Jiangshui*. The strain was identified as *Lactobacillus paracasei* and designated as LAB 815. *Saccharomyces cerevisiae* strain [SC 8(3)] was selected from the laboratory-preserved yeasts as the most suitable aroma-producing yeast for co-fermenting Jerusalem artichoke (JA) *Jiangshui* with LAB 815. *In vitro* assays of hypoglycemic and uric acid-lowering abilities, together with antioxidant activity against free radicals (DPPH, -OH and superoxide anion), revealed that LAB strain exerted inhibitory effects on α-amylase, α-glucosidase, glucose dialysis delay index, xanthine oxidase, DPPH radicals, hydroxyl radicals and superoxide anion radicals, with inhibition rates of 61.79, 53.26, 55.67, 83.46, 96.64, 88.76, and 79.06%, respectively. Gastrointestinal fluid simulation experiments demonstrated that cooperation between LAB 815 and SC 8(3) markedly mitigated the adverse effects of the highly acidic gastrointestinal environment. Antimicrobial assays showed that JA *Ji*ang*shui* significantly inhibited the growth of several spoilage bacteria. These results indicate that co-fermentation of JA tubers with LAB 815 and SC 8(3) to produce JA *Jiangshui* confers significant health benefits and represents a promising approach for managing diabetes and hyperuricemia.

## 1 Introduction

Lactic acid bacteria (LAB) are probiotics that can convert sugars into lactic acid through fermentation, widely distributed in natural ecosystems, and exhibit high abundance, especially in animal-derived food matrices, with dairy products and fermented foods constituting their core natural reservoir (Miranda et al., 2021; Gephart et al., 2024). Owing to the physiological properties of lactic acid produced through sugar metabolism, these microorganisms demonstrate a wide range of biological functions that play a crucial role in supporting human health. In the traditional fermentation process, LAB significantly improves the quality of fermented food through metabolic regulation (Widyastuti et al., 2021). Taking dairy fermentation system as an example, LAB can increase the production of aroma compounds and improve antibacterial activity (Marcia et al., 2019; Oskar et al., 2020). Notably, the fermentation activity of LAB has been shown to improve abnormal blood glucose levels by modulating the complex interaction network between the gut microbiota and the host (Grom et al., 2020). As the core biocatalyst of the fermented food industry, LAB plays a key role in traditional fermentation systems such as dairy products (yogurt, cheese), plant-based fermented foods (pickles, sauerkraut, *Jiangshui*) (Li et al., 2014; Huang and Xiao, 2019; Wu et al., 2019; Zhao et al., 2020; Zhao H. X. et al., 2022). In this context, the screening of LAB strains with specific health benefits and the development of precise targeted starter formulations have become an important direction for the upgrading of modern fermented food industry.

Recently, the use of probiotic fermented foods has attracted increasing attention as a natural and sustainable way to manage and treat diabetes mellitus and hyperuricemia (Grom et al., 2020; Wang et al., 2023). Du et al. (2024) found that the fermentation process can effectively promote the production of active substances and enhance the antioxidant, hypoglycemic and antihypertensive activities of laver by using Monascus, compound yeast and LAB to ferment laver. The study of Modi et al. (2023) found that LAB fermented Rumex Indian beverage had protective effects on chronic alcohol-induced liver injury and diabetes in rats and hypoglycemic effects. Previous studies have also found that probiotic GR-3 yogurt significantly reduced serum uric acid levels and promoted uric acid excretion in feces and urine (Zhao S. et al., 2022).

*Jiangshui* is a kind of serofluid dish, which is the most popular traditional salt-free fermented vegetable in northwest China (Hu et al., 2023). It has a unique flavor and can be made from a variety of vegetables and contains a variety of nutrients and physiological functions (Hou et al., 2015). Such as stimulate appetite, promote digestion, reduce the risk of heat stroke, lower cholesterol, blood sugar, uric acid and blood pressure (Li et al., 2017; Khan et al., 2023). It has been stated that LAB are predominant microorganisms in *Jiangshui* (Jin et al., 2024). Studies have shown that LAB originated from *Jiangshui* possess excellent antioxidant activity (Hu et al., 2023; Ren et al., 2023), and could attenuate the hyperuricemia (Wu Y. et al., 2021). Moreover, Zhao H. X. et al. (2022) reported that the content of bioactive substances such as polysaccharides, betaine and total flavonoids was significantly changed after fermentation in wolfberry *Jiangshui* used LAB. Thus, the development of *Jiangshui*-originated LAB with high functional efficiency as fermentation bacteria has a very broad prospect.

Jerusalem artichoke (*Helianthus tuberosus*, JA) is a perennial plant from the sunflower family Asteraceae natived to North America and was introduced to China at the end of the 18th century (Zhao 2020). A previous studies reported that JA is a highly nutritious vegetable with numerous health benefits. It may aid in the management of cardiovascular disease, chronic infectins, chronic fatigue syndrome and immune system disorders (Reshetnik, 2011; Sawicka et al., 2018; Michalska-Ciechanowska et al., 2019). It also can reduce high cholesterol, triglycerides, uric acid and high glucose levels (Yildiz et al., 2006; Petkova et al., 2014; Catană et al., 2018). Additionally, JA helps with weight loss, protect the gastric mucosa and prevent constipation (Sawicka, 2016; Nizioł-Łukaszewska et al., 2018; Eunson et al., 2019). JA is rich in inulin, it is an indigestible and biodegradable natural functional dietary fiber, which plays a dominant role in the function of JA as a high-quality natural prebiotic (Wang and Li, 2022). LAB fermentation can degrade inulin, making it easier to digest. The suitable LAB strain system is crucial in enhancing the functional and digestibility of fermented JA. Previous studies have shown that the fermentation of JA by Lactobacillus and yeast not only improves its nutritional value, but also produces bioactive compounds with potential therapeutic effects in diabetes and hyperuricemia (Lai et al., 2015; Du et al., 2024). Therefore, utilizing Jerusalem artichoke (JA) as a fermentation substrate in combination with lactic acid bacteria and yeast presents a novel approach for producing JA *Jiangshui*—a functional fermented beverage. This strategy not only enhances the nutritional profile and flavor of the drink but may also improve its metabolic health benefits, including support for gut microbiota, immune modulation, and improved glucose metabolism. The synergistic action of probiotics and JA’s bioactive compounds opens new possibilities for developing health-promoting functional beverages.

The current research primarily focuses on the targeted regulation of single strain or simple microbial communities to influence the quality indices of fermented *Jiangshui*. However, there remains a significant gap in understanding the transformation mechanisms of bioactive compounds within the Jerusalem artichoke (JA) matrix during fermentation by complex mixed microbial systems. To address these gaps, the present study aimed to (i) Screening and identification of *Jiangshui*-originated lactic acid bacteria with high hypoglycemic properties; (ii) Investigate the functional properties and fermentation performance of JA *Jiangshui* by using Lactobacillus and yeast and evaluate its effects on blood glucose and uric acid levels; and (iii) provide a scientific basis for the development of new dietary strategies to prevent and manage metabolic diseases and to develop a functional JA-based fermented beverage with potential metabolic health benefits through the selection of high-performing probiotic strains and evaluation of their hypoglycemic effects.

## 2 Materials and methods

### 2.1 Isolation of LAB from *Jiangshui*

Three kinds of *Jiangshui* sample were purchased from Gansu Longjiangyuan Agricultural Science and Technology Co., Ltd., including Tianshui *Jiangshui* pickles. A representative *Jiangshui* sample (1 g, each) was taken and diluted to 10^−7^ in a 10-fold serial gradient using sterile 0.85% normal saline (Chengdu Cologne Chemical Co., Ltd., Chengdu, China) as a diluent. These dilutions (100 μL, each) were injected into the culture dish, and then poured into 15–20 mL MRS (Beijing Soleibao Technology Co., Ltd., Beijing, China)-CaCO_3_ (Laiyang Shuangshuang Chemical Co., Ltd., Shandong, China) agar (Beijing Soleibao Technology Co., Ltd., Beijing, China) at about 50 °C, mixed, cooled and solidified, inoculation and cultured at 30 °C for 72 h. According to the colony size, shape, color and the size of the calcium ring, single colonies were selected and streaked onto MRS solid medium and cultured at 30 °C for 72 h. Pure colonies were obtained by repeatedly streaking and purifying 2–3 times on MRS solid medium and microscopic examination. Gram staining and catalase test were performed on all isolated and purified strains. If Gram staining was positive and catalase contact test was negative, it could be initially identified as LAB. Isolated colonies were cultured in MRS broth, incubated at 30 °C for 24 h, and harvested by centrifugation (TGL-16G centrifuge, Shanghai, China) for 5 min at 7,656 × g for preparing a frozen stock. Briefly, cell pellets were suspended in MRS broth (Oxoid) containing 25% glycerol, and the stock cultures were stored at −80 °C until use (Zhao H. X. et al., 2022; Gephart et al., 2024).

### 2.2 Screening of hypoglycemic functional LAB

#### 2.2.1 Preparation of cell-free supernatants

The frozen LAB strains were streaked on MRS agar plate and cultured at 30 °C for 48 h. Each lactic acid bacteria on the plate was inoculated in MRS broth, cultured at 30 °C for 24 h, and centrifuged at 804 × g for 15 min to remove bacteria. The pH of the supernatant was adjusted to 7.4 using 1 M NaOH and passed through a 0.22 μm membrane filter to obtain cell-free supernatant (Muganga et al., 2015).

#### 2.2.2 α-glucosidase inhibitory activity

The inhibition of α-glucosidase was determined using an assay modified from Ankolekar et al. (2012). α-Glucosidase (Beijing Soleibao Technology Co., Ltd., Beijing, China) was assayed by combining 50 μL sample extract and 100 μL α-glucosidase (1 U/mL) in PBS (0.1 M, pH 7.4), and incubating at 37 °C for 10 min. Next, 50 μL 5 mmol/L PNPG (Beijing Soleibao Technology Co., Ltd., Beijing, China) solution was added to each well, and the reaction mixture was incubated at 37 °C for 10 min. The absorbance at 405 nm (OD_405_) before and after the reaction was determined using a microplate reader, and 50 μL PBS was used in place of sample extract as the control. Acarbose (1 mg/mL) was a positive control in alpha-glucosidase inhibitory assay. The α-glucosidase inhibitory activity was calculated according to the Equation (1):


(1) Inhibition(%)=1−As/Ac×100

Where, As is the OD_405_ of the sample, and Ac is the OD_405_ of the control.

#### 2.2.3 α-amylase inhibitory activity

α-amylase inhibitory activity of the strains was evaluated as described by Vankudre et al. (2015). Briefly, For the determination of α-amylase (Beijing Suolaibao Technology Co., Ltd., Beijing, China), 250 μL of cell-free supernatants (CFS) was added to 250 μL of α-amylase solution (0.5 mg/mL) and pre-incubated at 25 °C for 10 min. The reaction mixture was then incubated with 250 μL of starch solution (1% w/v in 0.02 M sodium phosphate buffer) at 25 °C for 10 min. Next, the reaction was terminated with the addition of 500 μL of DNS color reagent (96 mM DNS and 5.31 M sodium potassium tartrate in 2 M sodium hydroxide solution). The reaction mixture was then boiled for 5 min, allowed to cool, and diluted four-fold with water. The absorbance was measured at 540 nm. The inhibition rate was calculated according to the Equation (2):


(2) Inhibition(%)=[(A−B)/A]×100

Where, A is the absorbance of the control, B is the absorbance of the sample.

#### 2.2.4 Screening of high hypoglycemic LAB

Three LAB strains with the highest hypoglycemic activity were selected to ferment JA *Jiangshui* with 10% (v/v) inoculation amount and cultured at 30 °C for 3 days. The inhibition rates of two enzymes, i.e., α-glucosidase and α-amylase were determined by sampling. The LAB strain with the highest inhibition rate of the two enzyme activities was selected.

### 2.3 Screening of aroma-producing yeast

Four yeast strains were used: *Pichia pastoris* 7(6), *Saccharomyces cerevisiae* 8(3), *Saccharomyces cerevisiae* 811, *Hansenula polymorpha* 9(6). They were provided by the Fermentation Engineering Laboratory of Lanzhou Jiaotong University. Each strain was cultured in YPD medium with 200 g/L glucose at 25 °C, with shaking 120 rpm for 96 h. Each strain was combined with high hypoglycemic LAB to ferment JA *Jiangshui*. The strains were screened by measuring the total acid content and total ester content of JA *Jiangshui* after fermentation. The total acid content and total ester content were determined as follows:

The method of measuring the total acid content (titratable acidity) was titration to the endpoint of pH 8.2 with 0.05 M NaOH (Wu et al., 2012). And total ester content was evaluated as described by Chen et al. (2023). Fifty milliliter YPD broth was added to 150 mL conical flask. After sterilization, 1 × 10^7^ yeast cells were inoculated at 28 °C for 72 h, and the product represented the fermentation broth. Then, 25 mL of fermentation broth was placed in a 250 mL conical flask, 25 mL of distilled water and two drops of phenolphthalein were added, and titrated to pink with 0.1 mol/L NaOH solution. Subsequently, 15 mL 0.1 mol/L NaOH solution was added and saponified at 40 °C for 24 h. Reverse titration was carried out with 0.1 mol/L HCl solution until the red color disappeared completely. The volume of HCl consumed was recorded and the blank medium (PBS) was used as a control. The ability to produce esters is calculated according to the following Equation (3):


(3) X(g/L)=[C×(Va−Vb)×88]/25=3.52×C×(Va−Vb)

Where, X is the concentration of total ester (g/L), C is the actual con centration of the hydrochloric acid solution (mol/L), Va is the volume of hydrochloric acid consumed by the sample in the blank control (mL), Vb is the volume of hydrochloric acid consumed by the sample (mL), 88 is the molar mass fraction of ethyl acetate, g/mol; and 25.00 is the sample volume (mL).

### 2.4 Strain identification

Identification of LAB: The supernatant of 12 h culture of 1.0 mL LAB strain was added to 1.5 mL EP tube and centrifuged at 12,000 r/min for 5 min. The DNA of the strain was extracted by DNA rapid extraction kit, and the 16 S rDNA universal primers were used for PCR amplification. The forward primer was 27F (5′-AGAGTTTGATCCTGGCTCAG-3′) and the reverse primer was 1492R (5′-TACGGYTACCTTTGTTACGACTT-3′) (Garcia et al., 2016).

Identification of yeast: The strains were identified using sequence analysis of the 5.8S rDNA domain, where amplification was performed using Internal Transcribed Spacer (ITS), ITS1 (5′-TCCGTAGGTGAACCTGCGG-3′) and ITS4 (5′-TCCTCCGCTTATTGATATGC-3′) (Lai et al., 2022). The DNA sequencing analysis was carried out by Shanghai Shenggong Bioengineering Technology Service Co., Ltd. (Shanghai, China). The DNA sequences comparisons were analyzed with Basic Local Alignment Search Tool (BLAST) software (National Center for Biotechnology Information, MD, United States). Pure strain cultures were stored using MRS broth (Oxoid, UK) supplemented with 20–30% glycerol (Oxoid-UK) at −80 °C.

### 2.5 Acid and bile salt tolerance

The method of Son et al. (2017) was modified to evaluate the tolerance of each LAB strain to acid and bile salts. Highly hypoglycemic LAB were grown in 10 mL MRS broth at 37 °C for 18 h, and aroma-producing yeasts were grown in 10 mL YPD broth at 25 °C for 96 h. The activated bacterial solution was centrifuged at 12,000 r/min for 5 min, and the bacterial precipitate was collected. After washing twice with PBS, 1 mL LAB suspension (2 × 10^8^ CFU/mL) and 1 mL yeast suspension (2 × 10^8^ CFU/mL) was suspended in 9 mL MRS broth (pH 2) and YPD broth (pH 2), or suspended in 9 mL MRS broth [0.3% (w/v) bile salt (China Damao Chemical Reagent Factory) and YPD broth (0.3% (w/v) bile salt), and incubated at 37 °C for 3 h]. Among them, 1 mL of the bacterial suspension was added to 9 mL of the culture medium, and the pH did not change significantly and remained around 2. The bacterial suspensions at 0 and 3 h were counted to calculate the survival rate using Equation (4), where N0 and N represent the initial and final cell numbers, respectively.


(4) Survival rate(%)=LgN/LgN0×100

Where, N0 is the initial viable count of lactic acid bacteria (LAB) or yeast before exposure in an acidic MRS broth (pH 2) containing 0.3% (w/v) bile salt (CFU/mL), N is the number of viable bacteria after 3 h of culture at 37 °C under the same stress conditions (CFU/mL).

### 2.6 Adhesion

#### 2.6.1 Hydrophobicity

The cell surface hydrophobicity of LAB strains was evaluated using a slightly modified microbial hydrocarbon adhesion method (Ren et al., 2014). The LAB strains were cultured in MRS broth at 37 °C for 18 h, and the yeast strains were cultured in YPD broth at 25 °C for 96 h. The bacteria were collected by centrifugation at 6,000 × g for 15 min. Each strain was washed twice with sterile phosphate buffered saline (PBS, 0.1 M, p H 7.4) containing 0.80% NaCl, 0.02% KCl, 0.02% KH_2_PO_4_ and 0.22% disodium hydrogen phosphate, and suspended in 0.1 M KNO_3_ to an optical density (OD_600_) of 0.5 ± 0.05 (OD_Initial_). 1.0 m L xylene was added to 3.0 m L suspension, pre-incubated at 37 °C for 10 min, vortexed for 2 min, and incubated at 37 °C for 20 min. The two phases (water and xylene) were separated. The aqueous phase was collected and its OD_600_ (OD_Time_) was measured. Hydrophobicity is calculated according to the following Equation (5):


(5) Hydrophobicity(%)=1−(ODTime/ODInitial)×100

Where, OD_Initia_ is the optical density (OD_600_) of the bacterial suspension at 600 nm of 0.1 M KNO_3_ before the addition of xylene, representing the initial cell concentration, OD_Time_ is OD_600_ after the water phase and xylene are mixed and separated, which reflects the interaction between the remaining cells in the water phase and the hydrocarbon.

#### 2.6.2 Auto-aggregation

The method of Xu et al. (2009) was modified to evaluate the self-aggregation of LAB. The LAB strains were cultured in MRS broth at 37 °C for 18 h, and the yeast strains were cultured in YPD broth at 25 °C for 96 h. The bacteria were collected by centrifugation at 6,000 × g for 15 min. Each strain was washed twice with PBS (0.1 M, pH 7.4) and resuspended in PBS to OD_600_ = 0.5 ± 0.05 (OD_Initial_). Then, 2 mL suspension was vortexed for 10 s and incubated at 37 °C for 2 h. The upper suspension was collected and its OD_600_ (OD_Time_) was measured. Calculate the degree of automatic aggregation according to the Equation (6):


(6) Auto−aggregation(%)=1−(ODTime/ODInitial)×100

Where, OD_Initia_ is the optical density (OD_600_) of the bacterial suspension in phosphate buffer solution at 600 nm immediately after resuspension and vortex, reflecting the initial cell density. OD_Time_ is corresponding to OD_600_ of the suspension after incubation at 37 °C for 2 h, indicating that the remaining unaggregated cells in the aqueous phase.

### 2.7 Preparation of JA *Jiangshui*

JA is purchased from a store in Lanzhou, Gansu, China. The JA was washed and cut into filaments (5–7 cm). Sixty gram of JA was weighed and blanched with boiling water for 2 min. One hundred ninety-six milliliter of drinking water was added to 4 g of flour. After mixing well, it was boiled to make a noodle soup. The blanched JA was mixed with the noodle soup at a mass ratio of 3:10 and put into a jar. The screened high hypoglycemic LAB were cultured in MRS medium at 37 °C for 18 h, and the aroma-producing yeast was cultured in YPD medium at 25 °C for 96 h. The viable count of the bacterial suspension to 1 × 10^7^ CFU/mL and then inoculated it into 200 mL of noodle soup at a volume ratio of 1:1 (high-hypoglycemic LAB: aroma-producing yeast). The inoculum volume was 16% (v/v), and the mixture was fermented at 30 °C for 3 days.

### 2.8 Hypoglycemic and uric acid-lowering effects of JA *Jiangshui in vitro*

The cell-free supernatant of JA *Jiangshui* was collected at 4 °C and 6,000 × g for 15 min. The α-glucosidase inhibitory activity was calculated using Equation 1 and expressed as a percentage. The inhibitory activity of α-amylase was calculated using Equation 2 and expressed as a percentage. Acarbose (1 mg/mL) was a positive control in the determination of α-amylase and α-glucosidase inhibition.

The method of was Zheng et al. (2021) modified to evaluate the glucose dialysis retardation index (GDRI). One milliliter of sample was added to 10 mL of 0.1 mol/L glucose solution, shaken continuously at 37 °C for 1 h, and then transferred to a dialysis bag with a molecular weight cut-off value of 7,000. At the same time, blank control samples (containing glucose, without sample) and sample control samples (containing sample, without glucose) were prepared. The dialysis bag was placed in a 500 mL beaker, oscillated at 37 °C for 60 min, and 2 mL of glucose dialysate was taken every 30 min. The glucose concentration was measured and calculate the GDRI according to the Equation (7):


(7) GDRI(%)=[1−(A1−A2)/A3]×100

Where, A1 is the glucose concentration of the sample solution; A2 was used as the glucose concentration of the sample control; A3 was the glucose concentration of the blank control.

The ability of strains to inhibit xanthine oxidase (XO) is as follows (Chen et al., 2017). The JA *Jiangshui* was centrifuged at 10,000 r/min for 10 min to obtain a cell-free supernatant for XO inhibitory activity analysis. The enzyme reaction system was prepared according to Table 1. The absorbance was measured at 293 nm at 0 and 10 min of reaction time. The inhibition rate of JA *Jiangshui* on XO was calculated according to the following Equation (8):


(8) $$\mathrm{XO}\;\text{Inhibition rate}=[1-(\mathrm{As}-\mathrm{As}0)/(A_{b}-A_{b}0)]\times 100\%$$

**Table 1:** Reaction system for determination of xanthine oxidase inhibition rate.

Groups	PBS buffer (μL)	Xanthine oxidase (μL)	Allopurinol (μL)	CFE of the strain (μL)	Xanthine (μL)
Sample	140	20	0	20	20
Positive control	140	20	20	0	20
Blank control	160	20	0	0	20

Where, As0 and As are the absorbance value at reaction time of 0 min and 10 min of sample and positive control groups, respectively. A_b_0 and A_b_ are the absorbance value at reaction time of 0 min and 10 min of blank control, respectively.

The vitro hypoglycemic and uric acid-lowering abilities of JA *Jiangshui* fermented by different fermentation methods (LAB fermented JA *Jiangshui*, LAB and yeast mixed fermented JA *Jiangshui*, JA *Jiangshui* fermented with traditional starter culture (*Jiangshui* Yinzi) and natural fermentation of JA tuber) were determined, and JA juice was used as a control.

### 2.9 Tolerance to simulated gastric and intestinal fluid

Simulated gastrointestinal digestion was performed according to the method of Cruz-Huerta et al. (2015) with modifications. An amount of 6 mg of the separated components was added to 20 mL of simulated gastric fluid (1 g pepsin was dissolved in 100 mL of deionizedwater, and 1.64 mL of 0.1 mol/L HCl was added). The samples were incubated for 180 min at 37 °C. The reactions were stopped by heating at 80 °C for 5 min, and the inhibitory activities of α-glucosidase, α-amylase and xanthine oxidase were determined. In 20 mL simulated intestinal fluid (0.68 g K_2_HPO_4_ and 1 g trypsin were dissolved in 100 mL deionized water, and the pH value was adjusted to 6.8 with dilute NaOH solution). It was heated at 37 °C for 180 min. The inhibitory activities of α-glucosidase, α-amylase and xanthine oxidase were determined after the reaction was terminated by heating. All inhibition rates were measured in triplicate.

The tolerance of the JA *Jiangshui* fermented by different fermentation methods (LAB fermented JA *Jiangshui,* LAB and yeast mixed fermented JA *Jiangshui, Jiangshui* Yinzi and natural fermentation of JA tubers) to simulated gastric and intestinal fluid were determined, and JA juice was used as a control.

### 2.10 Antioxidant activity

#### 2.10.1 DPPH (2, 2-diphenyl-1-picrylhydrazyl) radical scavenging activity (DPPH RSA)

The DPPH radical scavenging activity of the samples was examined according to the procedure described by Xiao et al. (2014). The sample solution was mixed with the working solution and stored in the dark at 25 °C for 30 min. The absorbance of the sample was then measured at 514 nm to calculate the scavenging capacity. The DPPH free radical scavenging capacity was calculated using the following Equation (9):


(9) DPPHRSA(%)=[1−(Asample−Ablank)/ADPPH]×100%

Where, A_sample_ is the absorbance in the presence of samples, A_blank_ is the absorbance in the absence of samples, and A_DPPH_ is the absorbance of the DPPH solution.

#### 2.10.2 Hydroxyl radical scavenging activity

The Hydroxyl radical scavenging ability assay was performed using the method previously reported by Ozdal et al. (2019) with slight modifications. The Fenton solution was quickly mixed with the sample treatment solution. Following a reaction at 37 °C for 1 min, 2 mL of a Griess reagent was introduced, and the absorbance was measured at 550 nm at room temperature for 20 min. To assess the hydroxyl radical scavenging capability, the following Equation (10) was utilized.


(10) $$\begin{array}{l} \text{HRSA}\;(\%)=(\text{Acontrol}-\text{Asample})\\ /(\text{Acontrol}-\text{Ablank})\times 100\% \end{array}$$

Where, A_control_ is the absorbance of deionized water instead of samples, A_sample_ is the absorbance in the presence of samples, and A_blank_ is the absorbance in the absence of samples.

#### 2.10.3 Superoxide anion radical scavenging activity

The ability to scavenge superoxide anion radicals was measured using the previously reported method by Zhang et al. (2021) with minor modifications. 4.5 mL of 0.05 mol/L Tris–HCl buffer (pH 8.2) in a test tube with a stopper was placed in a water bath at 25 °C for 10 min. Subsequently, the solution was combined with 1 mL of samples and 0.4 mL of 6 mmol/L pyrocatechol, mixed, and allowed to stand for 4 min, and then 1 mL of hydrochloric acid at a concentration of 8 mol/L was added to terminate the reaction. The absorbance of the mixture was measured at 320 nm. The superoxide anion radical scavenging activity (SARSA) was determined using the following Equation (11):


(11) $$\begin{array}{l} \text{SARSA}\;(\%)=[\text{Acontrol}-(\text{Asample}/\text{Astandards})]\\ /A_{\text{control}}\times 100\% \end{array}$$

Where, A_control_ is the absorbance of deionized water instead of samples, A_sample_ is the absorbance in the presence of samples, and A_standards_ is the absorbance of the VC standard solution instead of samples.

### 2.11 Antimicrobial activity assays

The Oxford Cup method is used according to this method, with minor modifications (Riaz Rajoka et al., 2017). The antibacterial activity of JA *Jiangshui* was tested with *Staphylococcus aureus*, *Escherichia coli* and *Klebsiella pneumoniae* as indicator bacteria. The antibacterial effect was evaluated according to the diameter of the inhibition zone, and the average value of 3 replicates was taken. The LAB fermented JA *Jiangshui*, *Jiangshui* Yinzi and natural fermentation of JA tubers were used as controls.

### 2.12 Determination of volatile compounds

Volatile compounds were determined according to the method modified by Sevindik et al. (2022), which were detected by a gas chromatograph (Agilent 7890A, United States) with a mass selective detector (MSD) (Agilent 5975C, United States). Samples (5 mL) and NaCl (2 g) were added to headspace bottles. A HP-INNOWAX capillary column (0.25 mm × 30 m ID, 0.25 μm film thickness) was selected to separate volatile compounds. After holding at 60 °C for 3 min, the column temperature was increased to 230 °C at a rate of 2 °C/min and then increased to 245 °C at a rate of 3 °C/min and kept at 245 °C for 20 min. Helium was used as a carrier at a flow rate of 1 mL/min. The volatile compounds were identified by mass spectrometry in comparison of the NIST10 database. The concentration of volatile compounds was determined by using an in-ternal standard (2-octanol).

### 2.13 Analysis of free amino acids

The content to free amino acids in *Jiangshui* was measured using the previously reported method by Park et al. (2024). The *Jiangshui* sample was centrifuged at 12,000 × g at 4 °C for 10 min to remove solid residues. The supernatant was filtered with 0.22 μm PVDF membrane to obtain the filtrate. The filtrate was then subjected to amino acid analysis using the JASCO LC-2000 Plus Series HPLC system (JASCO, Tokyo, Japan). Chromatographic separation was conducted with an AccQ-Tag Amino Acids C18 Column (3.9 mm × 150 mm, 4 μm; Waters Corporation, Milford, MA, United States). Gradient elution was carried out with AccQ-Tag/water (solvent A; 10:90, v/v) and acetonitrile/water (solvent B; 60:40, v/v). The following binary mobile phase linear gradients were used: 100% A at 0 min, 98% A at 5 min, 93% A at 15 min, 90% A at 19 min, 67% A at 32–33 min, 0% A at 34–37 min, and 100% A at 38 min. Injection volumes were 10 μL, and the column temperature and flow rate were 37 °C and 1 mL/min, respectively. The detection was performed using a fluorescent detector. AccQ-Fluor Reagent Kit (WAT052880, Waters Corporation, Milford, MA, United States) was simultaneously used as derivatizing agents, according to the manufacturer’s instructions. The photodiode array (PDA) detector (MD-2018 Plus, JASCO, Tokyo, Japan) wavelength was set to 254 nm for the determination of the AccQ-Fluor Reagent-derivatized amino acids. The concentrations ofindividual free amino acids were determined using five-point calibration curves ofthe amino acid standard (WAT088122, Waters Corporation, Milford, MA, United States). The free amino acid contents oftwo samples prepared in the same conditions were analyzed twice.

### 2.14 Analysis of the pH and cell growth of JA *Jiangshui*

The pH and cell growth of JA *Jiangshui* according to the method of Li et al. (2023). The pH was measured by a Pen pH detector (Dongguan Wanchuang Electronic Products Co., Ltd.). The plate count method was used for counting. According to the dilution factor and the number of colonies on the plate after culture at 30 °C (30–300), the plate was incubated at 30 °C for 48 h, and the number of colony forming units (CFU/mL) of high-sugar LAB and aroma-producing yeast was calculated.

### 2.15 Statistical analysis

Taxonomic strain identification was performed by comparing the sequences of each screen with the 16S rDNA and ITS rDNA gene sequences available in the NCBI databases by a basic BLAST search. A distance matrix and phylogenetic tree were generated for all representative isolates of the molds and yeasts using the neighbor-joining method in MEGA (version 11.0, Mega Limited, Auckland, New Zealand) software. Statistical analysis was performed using the IBM SPSS Statistics (version 27.0; one-way ANOVA determination in IBM Corp., Chicago, IL, USA) software package. ANOVA and Tukey’s multiple range tests were applied to the data to determine significant differences, and the model was statistically significant with a value of *p* < 0.05. GraphPad Prism (version 9.5.0 GraphPad Software, San Diego, California, United States) software was used to process the data and draw graphics of volatile flavor compounds.

## 3 Results

### 3.1 Screening and identification of LAB with high hypoglycemic function

Fifty LAB isolates were initially obtained from *Jiangshui* sample, and nine isolates were preliminarily considered as LAB with high hypoglycemic function based on the inhibitory effects on the activities of α-amylase and α-glucosidase (Table 2). The inhibition rates of α-amylase and α-glucosidase of nine LAB fermentation supernatant were in the range of 10.23–40.11% and 9.86–42.50%, respectively. Based on a comprehensive comparison of α-amylase and α-glucosidase inhibition rates, three lactic acid bacteria (LAB) strains with the highest inhibitory activities were selected.

**Table 2:** Determination of hypoglycemic ability of lactic acid bacteria isolated from different aquatic products *in vitro*.

Strain	Inhibitory rates (%)	
	α-glucosidase	α-amylase
Acarbose (1 mg/mL)	80.66 ± 1.69^a^	78.17 ± 1.56^a^
752	18.23 ± 1.09^g^	20.33 ± 0.54^g^
763	9.86 ± 0.23^j^	27.33 ± 1.83^f^
815	42.50 ± 1.06^b^	40.11 ± 1.10^b^
818	23.50 ± 0.60^d^	36.93 ± 0.62^d^
833	26.80 ± 0.67^c^	39.27 ± 1.20^c^
954	21.50 ± 0.32^e^	30.89 ± 0.75^e^
963	20.24 ± 0.28^f^	10.23 ± 0.78^j^
1032	9.98 ± 0.42^i^	12.32 ± 0.52^i^
1049	15.27 ± 0.17^h^	18.36 ± 0.24^h^

All values in the table are mean ± standard deviation (*n* = 3). The mean values of different superscripts in the same column indicated significant differences between strains (*p* < 0.05).

**Figure 1:**
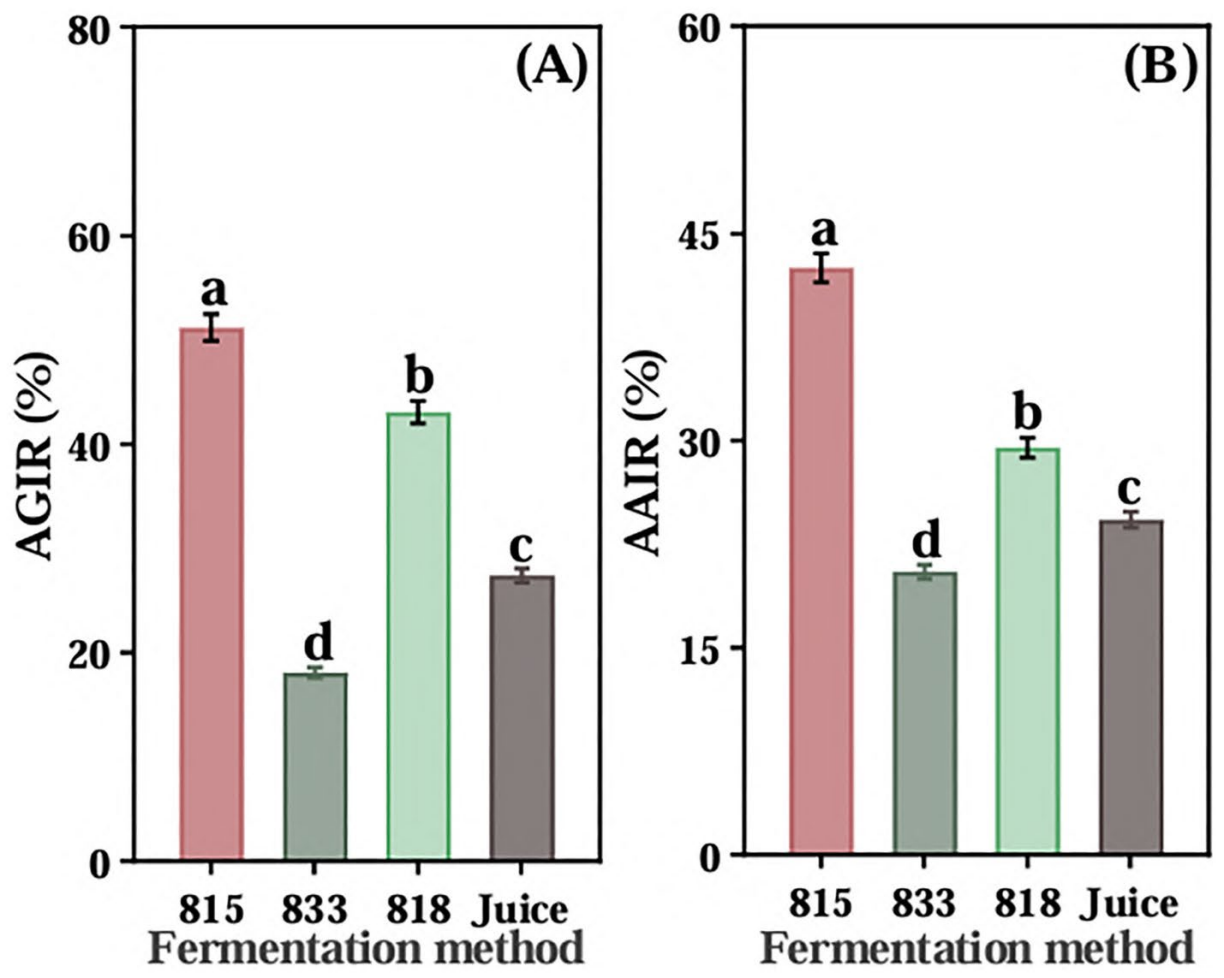
The inhibition rates of α-glucosidase **(A)** and α-amylase **(B)** of three kinds of high-sugar lactic acid bacteria (815,833,818) fermented JA *Jiangshui* and unfermented JA juice (Juice) were determined. AGIR is α-glucosidase inhibition rate; AAIR is α-amylase inhibition rate. All values in the figure were mean ± standard deviation (*n* = 3). There were significant differences in the values of different superscript letters (*p* < 0.05).

**Figure 2:**
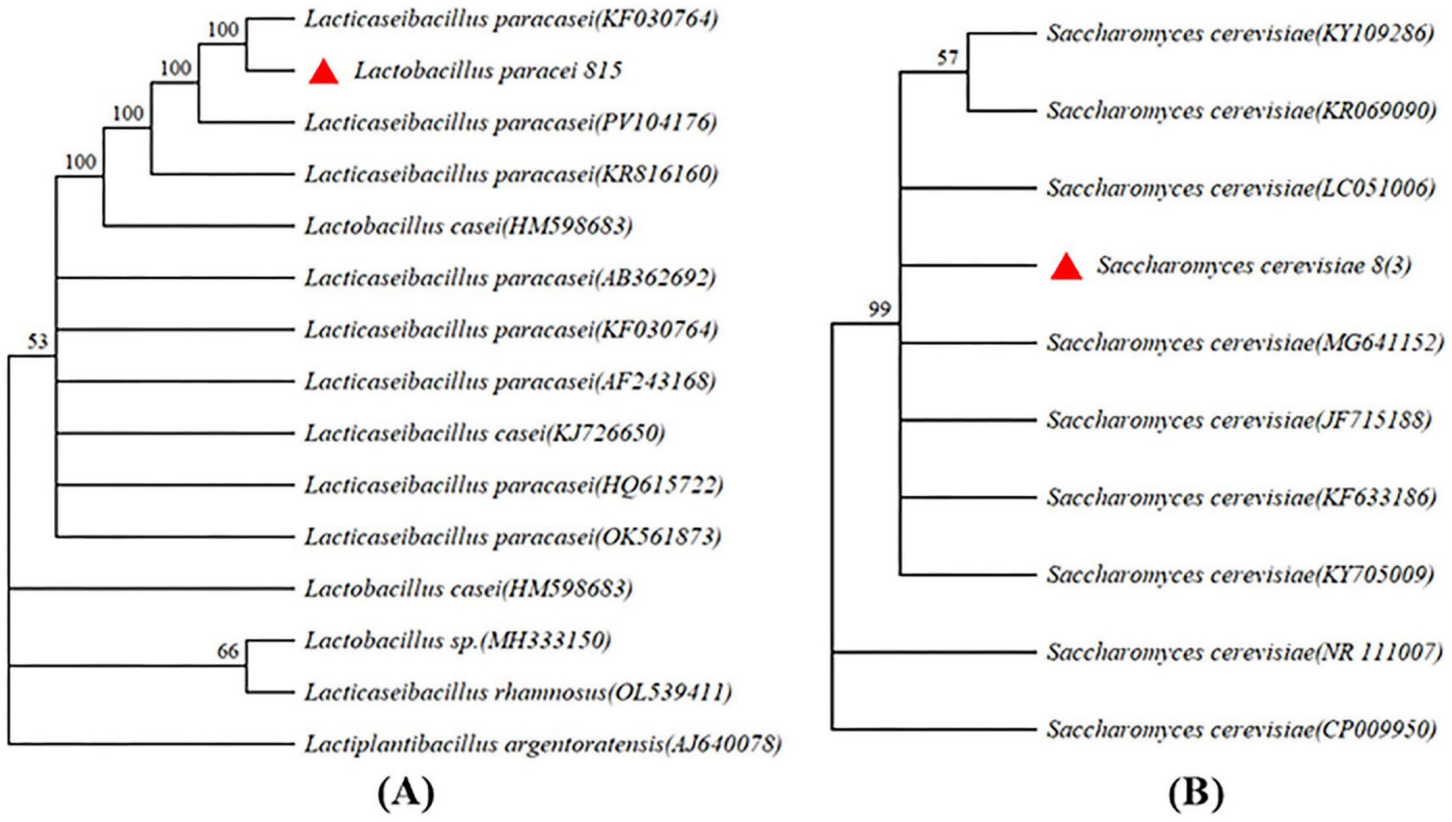
Phylogenetic tree was constructed using 16S rRNA gene sequence. **(A)** The correlation between the tested strains (*Lactobacillus paracasei* 815) and the members of Lactobacillus are shown in parentheses. The sequence of *Lactobacillus casei* (HM598683), *Lactobacillus* sp. (MH333150), *Lactobacillus rhamnosus* (OL539411) and *Lactobacillus argentoratensis* (AJ640078) were used as outgroups. **(B)** The correlation number between the tested strain [*Sacchammyces cerevisiae* 8(3)] and the members of the genus Saccharomyces are shown in parentheses. The sequences of *S. cerevisiae* (NR 111007) and *S. cerevisiae* (CP 009950) were used as outgroups.

The screened of three LAB were used to ferment JA *Jiangshui*. The inhibition rate of α-glucosidase of the JA *Jiangshui* fermented by LAB 815 and LAB 818 were significantly higher than that of JA juice (Figure 1A), and the JA *Jiangshui* fermented by LAB 833 was significantly lower than that of JA juice (Figure 1A). Among them, the inhibition rate on α-glucosidase activity of JA *Jiangshui* fermented by the strain 815 and JA juice were 51.23 and 27.42%, respectively, increased by 23.81% compared with unfermented JA juice (Figure 1A). The α-amylase inhibition rate of JA *Jiangshui* fermented with LAB 815 and LAB 818 was significantly higher than that of unfermented JA juice, whereas fermentation with LAB 833 resulted in a significantly lower inhibition rate compared to JA juice (Figure 1B). Among them, the JA *Jiangshui* fermented by strain 815 exhibited an α-amylase inhibition rate of 42.50%, compared to 24.26% for the unfermented JA juice—an increase of 18.24% (Figure 1B). The LAB 815 had the highest inhibition rate on α-glucosidase and α-amylase activity. According to the results of phylogenetic tree constructed using 16S rRNA gene sequences, LAB 815 was confirmed as *Lactobacillus paracasei* with 99.8% sequence similarity to *Lactobacillus paracasei* (PV104176) (Figure 2A).

### 3.2 Screening and identification of aroma-producing yeast

**Figure 3:**
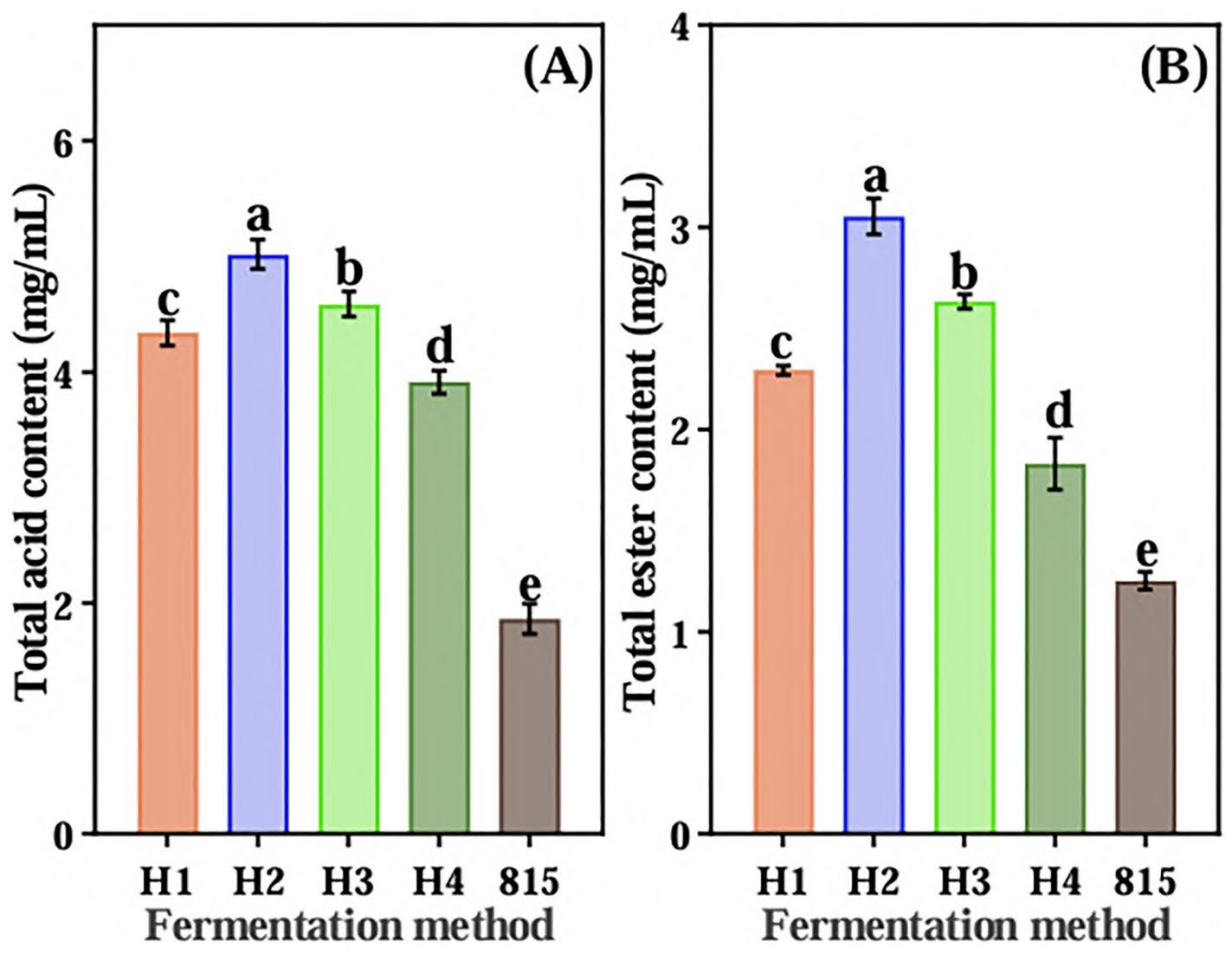
The total acid content **(A)** and total ester content **(B)** of JA *Jiangshui* co-fermented by four aroma-producing yeasts and high-sugar lactic acid bacteria 815 were determined. “HI,” “H2,” “H3,” and “H4” represented SC 7(6), SC 8(3), SC 811 or SC 9(2), respectively, in combination with LAB 815 to ferment JA *Jiangshui*. 815 is JA *Jiangshui* fermented by LAB 815. All values in the figure were mean ± standard deviation (*n* = 3). There were significant differences in the values of different superscript letters (*p* < 0.05).

Four aroma-producing yeasts were mixed with LAB 815 to ferment JA *Jiangshui*. The total acid content of JA *Jiangshui* fermented by the combination of four aroma-producing yeasts and LAB 815 was significantly higher than that fermented by LAB 815 alone (Figure 3A). JA *Jiangshui* fermented with the mixed strains 8(3) and 815 exhibited the highest total acid content, reaching 5.0171 mg/mL—an increase of 62.95% compared to fermentation with strain 815 alone. While, the total ester content of JA *Jiangshui* fermented by the combination of four aroma-producing yeasts and LAB 815 was significantly higher than that of JA *Jiangshui* fermented by LAB 815 alone (Figure 3B). The total ester content of JA *Jiangshui* fermented with the mixed strains 8(3) and 815 was also the highest, reaching 3.055 g/L, an increase of 58.98% compared to fermentation with strain 815 alone. These results indicated that the selected yeast strain 8 (3) was the most suitable for JA *Jiangshui* fermentation. Phylogenetic analysis based on 16S rRNA gene sequences identified the aroma-producing yeast 8(3) as *Saccharomyces cerevisiae* with 100% sequence similarity to *Saccharomyces cerevisiae* (KF633186) (Figure 2B).

### 3.3 Acid and bile salt tolerance

Our study showed that the survival rates of LAB 815 in acidic medium (pH 2) and bile salt medium (0.3% bile salt, 3 h) were 84.15 and 78.24%, respectively, after incubation for 3 h (Table 3). While, the survival rates of *S. cerevisiae* 8 (3) in acidic medium (pH 2) and bile salt medium (0.3% bile salt, 3 h) were 80.21 and 76.25%, respectively, after incubation for 3 h (Table 3). The survival rate of all strains in acidic medium was more than 80%, and was higher than in bile salt medium.

**Table 3:** The characteristics of probiotics (acid resistance, bile salt resistance, hydrophobicity, and self-aggregation ability) of *Lactobacillus paracasei* 815 and *Saccharomyces cerevisiae* 8(3) were determined.

Strains	Survival in gastric fluid (%)	Survival in intestinal fluid (%)	Hydrophobicity (%)	Auto-aggregation (%)
815	84.15 ± 4.56	78.24 ± 2.34	34.03 ± 1.56	27.24 ± 2.11
8 (3)	80.21 ± 3.73	76.25 ± 6.54	36.85 ± 3.25	20.88 ± 1.52

All values in the table were mean ± standard deviation (*n* = 3). The mean values of different superscripts in the same column indicated significant differences between strains (*p* < 0.05).

### 3.4 Adhesion

The adhesion characteristics of probiotics are an important factor in determining their probiotic properties. The cell surface hydrophobicity and self-aggregation of probiotics were positively correlated with adhesion. Our study shown that the hydrophobicity and self-aggregation of LAB 815 were 34.03 and 27.24%, respectively, and the hydrophobicity and self-aggregation of SC 8(3) were 36.85 and 20.88%, respectively (Table 3). The results showed that LAB 815 and SC 8(3) exhibited notable cell surface hydrophobicity and auto-aggregation ability, suggesting strong adhesion potential within the intestinal environment.

### 3.5 Hypoglycemic and uric acid-lowering ability *in vitro* of fermented JA *Jiangshui*

**Figure 4:**
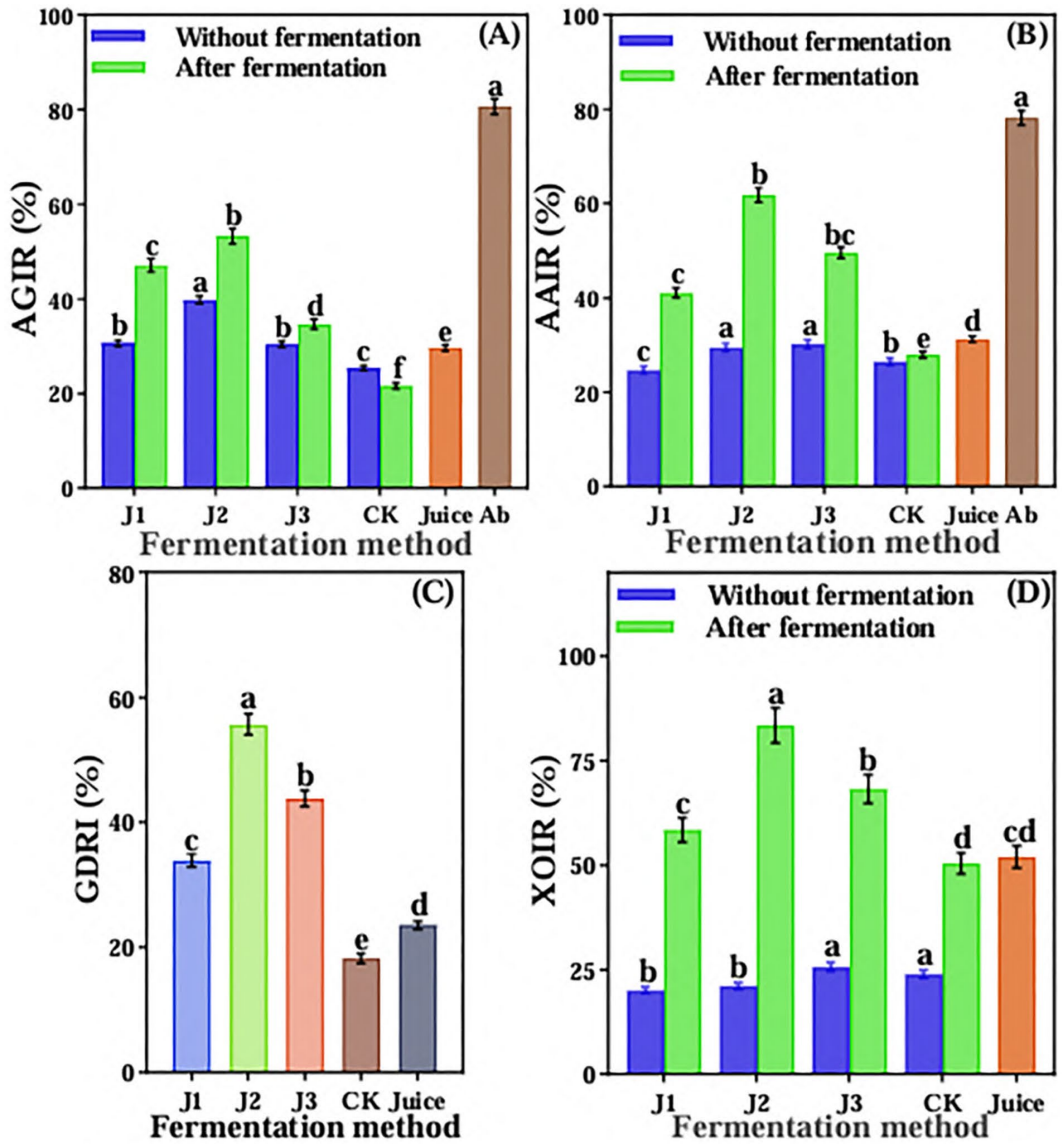
The α-glucosidase inhibition rate **(A)**, α-amylase inhibition rate **(B)**, glucose dialysis retardation index **(C)** and xanthine oxidase inhibition rate **(D)** of JA *Jiangshui* with different fermentation methods were studied. AGIR is α-glucosidase inhibition rate; AAIR is α-amylase inhibition rate; GDRI is Glucose dialysis retardation index; XOIR is xanthine oxidase inhibition rate; J1 is LAB 815 fermented JA *Jiangshui*; J2 is LAB 815 and SC 8(3) mixed fermented JA *Jiangshui*; J3 is JA *Jiangshui* fermented with traditional starter culture (*Jiangshui* Yinzi); CK is natural fermentation of JA tuber; Juice is JA juice; Ab is Acarbose. All values in the figure were mean standard deviation (*n* = 3). There were significant differences in the values of different superscript letters (*p* < 0.05).

Compared to pre-fermentation and JA Juice, the α-glucosidase inhibition rate significantly increased in J1, J2, and J3, whereas it significantly decreased in CK (Figure 4A). The J2 of post-fermentation had the highest inhibition rate of α-glucosidase, reaching 53.26%, which was 66.03% of acarbose. The inhibition rate of α-amylase also was significantly increased in J1, J2, and J3 compared with pre-fermentation and JA Juice, and there was no significant change observed in CK after fermentation as compared with pre-fermentation and JA Juice (Figure 4B). The J2 of post-fermentation had the highest inhibition rate of α-amylase, reaching 61.79%, which was 79.04% of acarbose. After fermentation, the glucose dialysis retardation index (GDRI) of J1, J2, and J3 increased significantly compared to unfermented JA juice, while the control (CK) showed a significant decrease (Figure 4C). The LAB 815 and SC 8(3) co-fermented JA *Jiangshui* had the greatest glucose dialysis retardation index, reaching 55.67%. The inhibition rate of xanthine oxidase of post-fermentation of JA *Jiangshui* was significantly increased than that of pre-fermentation, regardless of which fermentation method was adopted (Figure 4D). The inhibition rate of xanthine oxidase of J1, J2, and J3 were significantly higher than that of JA Juice, and there was no significant difference observed in the inhibition rate between CK and JA Juice. Among them, J2 had the highest inhibiting rate of xanthine oxidase, reaching 83.46%. The overall results showed that the ability of hypoglycemic and lowering uric acid *in vitro* of JA is enhanced after being fermented by probiotics to form JA *Jiangshui.*

### 3.6 Gastrointestinal fluid simulation of fermented JA *Jiangshui*

**Figure 5:**
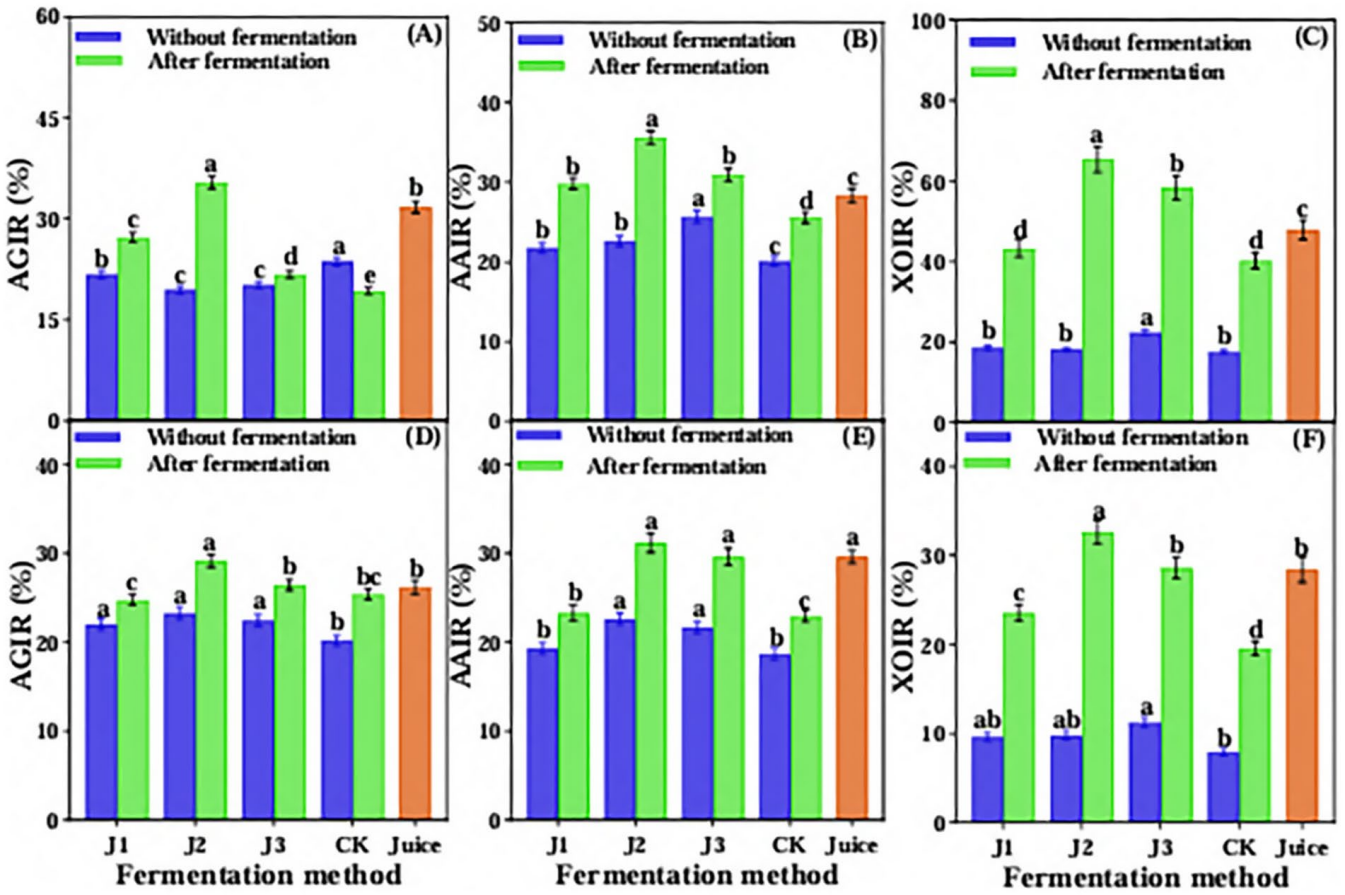
The inhibition rates of α-glucosidase **(A)**, a-amylase **(B)** and xanthine oxidase **(C)** *in vitro* gastric juice simulation and α-glucosidase **(D)**, α-amylase **(E)** and xanthine oxidase **(F)** in intestinal fluid simulation of JA *Jiangshui* with different fermentation methods were studied. AGIR is α-glucosidase inhibition rate; AAIR is α-amylase inhibition rate; XOIR is xanthine oxidase inhibition rate; J1 is LAB 815 fermented JA *Jiangshui*; J2 is LAB S15 and SC 8(3) mixed fermented JA *Jiangshui*; J3 is JA *Jiangshui* fermented with *Jiangshui* Yinzi; CK is natural fermentation of JA tuber; Juice is JA juice. All values in the figure were mean 4: standard deviation (*n* = 3). There were significant differences in the values of different superscript letters (*p* < 0.05).

The ability of JA *Jiangshui* fermented by different fermentation methods was evaluated for its ability to reduce blood glucose and uric acid in simulated gastrointestinal fluid *in vitro*. The results showed that the inhibition rates of α-glucosidase, α-amylase and xanthine oxidase changed in simulated gastric fluid *in vitro*. As shown in Figure 5A, the inhibition rate of α-glucosidase in J1, J2 and J3 was significantly increased compared with pre-fermentation, whereas a decrease was observed in the control (CK). After simulated gastric digestion, the post-fermentation sample J2 exhibited the highest α-glucosidase inhibition rate, reaching 35.42%, which was 3.7% higher than that of unfermented JA Juice. The inhibition rate of α-amylase and the inhibition rate of xanthine oxidase of JA *Jiangshui* were significantly increased than that of pre-fermentation. The J2 of post-fermentation had the highest inhibition rate of α-amylase and xanthine oxidase, reaching 35.64 and 65.38%, respectively, which were higher than JA Juice 7.29 and 17.48%. While, the CK had the lowest inhibition rate of α-amylase and xanthine oxidase and was lower than JA Juice after gastric fluid simulation (Figures 5B,C).

The results of JA *Jiangshui* fermented by different fermentation methods through intestinal fluid simulation showed that the inhibition rate of α-glucosidase, the inhibition rate of α-amylase and the inhibition rate of xanthine oxidase were significantly increased compared to pre-fermentation. The post-fermentation sample J2 exhibited the highest inhibition rates for α-glucosidase, α-amylase, and xanthine oxidase, reaching 29.19, 31.19, and 32.64%, respectively—significantly higher than the corresponding values in unfermented JA juice, which were 2.96, 1.58, and 4.24%. While, after intestinal fluid simulation, the J1 had the lowest inhibition rate of α-glucosidase, reaching 24.76%, which was lower than JA Juice 1.47%. The inhibition rate of α-amylase and xanthine oxidase were the lowest in CK, reaching 22.98 and 19.56%, respectively, which were lower than JA Juice 6.63 and 8.84% (Figures 5D–F). The overall results indicated that J2 had the highest inhibition rate of α-glucosidase, α-amylase and xanthine oxidase, and the ability to reduce blood glucose and uric acid *in vitro* was the best compared with other groups.

### 3.7 Antioxidant activity of fermented JA *Jiangshui*

**Figure 6:**
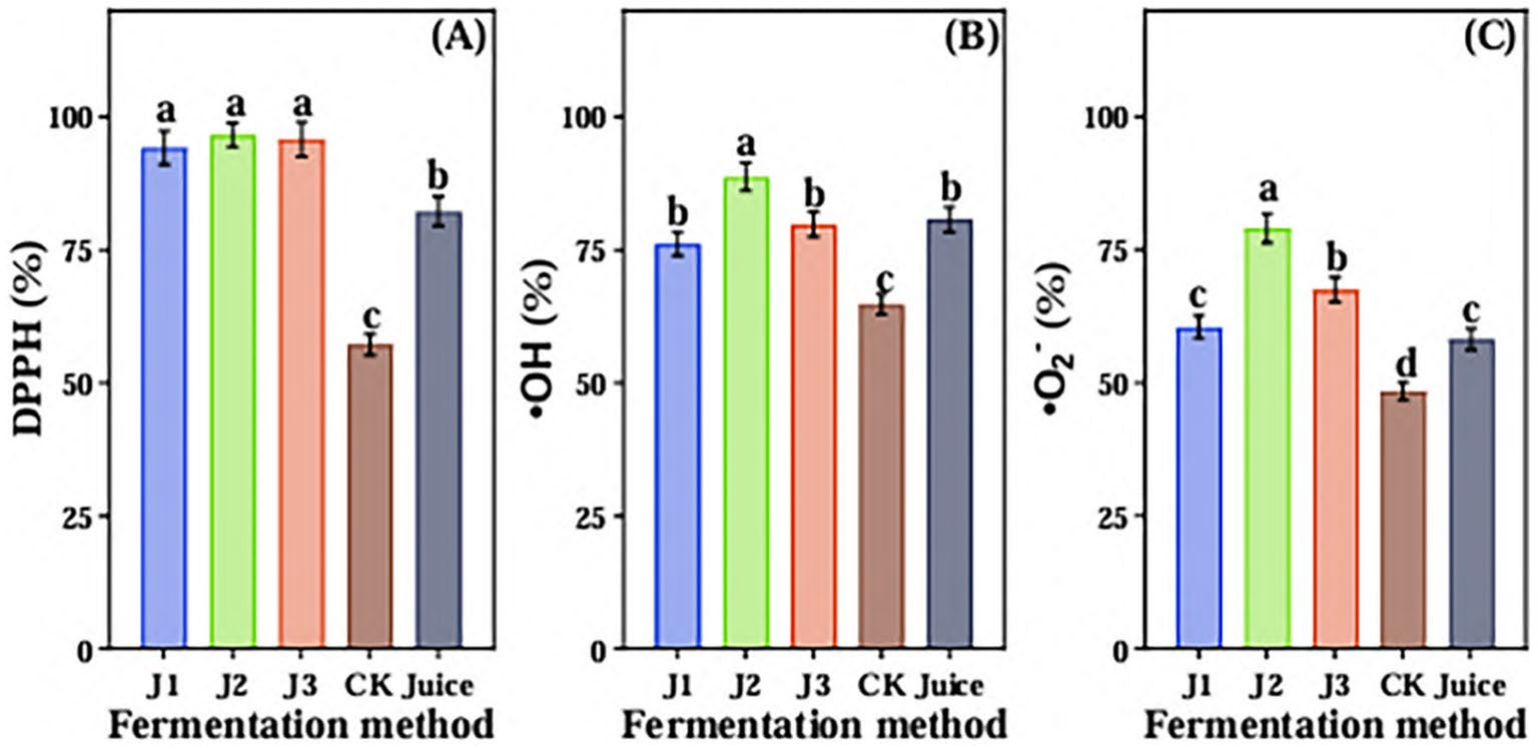
The scavenging ability of DPPH free radical **(A)**, hydroxyl radical **(B)** and superoxide anion radical **(C)** in Jerusalem artichoke *Jiangshui* with different fermentation methods was studied. DPPH is DPPH free radical scavenging rate; •OH is Hydroxyl radical scavenging rate; •O_2_: is Superoxide anion scavenging rate; J1 is LAB 815 fermented JA *Jiangshui*; J2 is LAB 815 and SC S(3) mixed fermented JA *Jiangshui*; J3 is JA *Jiangshui* fermented with *Jiangshui* Yinzi; CK is natural fermentation of JA tuber; Juice is JA juice. All values in the figure were mean ± standard deviation (*n* = 3). There were significant differences in the values of different superscript letters (*p* < 0.05).

The scavenging ability of DPPH free radical, hydroxyl radical and superoxide anion radical of JA *Jiangshui* by different fermentation methods was determined (Figure 6). As shown in Figure 6A, there was no difference between J1, J2 and J3 for DPPH free radical scavenging ability, and significantly higher than CK and JA Juice. The ability of scavenging DPPH free radicals was the lowest in CK. The DPPH free radical scavenging ability of J2, J3, J1, Juice and CK were 96.64 ± 2.28%, 95.83 ± 3.25%, 94.23 ± 3.2%, 82.32 ± 2.79% and 57.23 ± 1.94%, respectively. The hydroxyl radical scavenging ability of J2 was significantly higher than that of the other four groups. There was no difference between J1, J3 and Juice, and significantly higher than that of CK. The hydroxyl radical scavenging ability of J2, Juice, J3, J1 and CK were 88.76 ± 2.66%, 80.75 ± 2.42%, 79.83 ± 2.39%, 76.2 ± 2.28%, and 64.73 ± 1.94% (Figure 6B). The superoxide anion radical scavenging ability of J2 was significantly higher than other four groups, and there was no significant difference observed between J1 and Juice, and CK had the lowest ability of scavenging superoxide anion radical. The superoxide anion radical scavenging ability of J2, J3, J1, Juice and CK were 79.06 ± 2.76%, 67.51 ± 2.36%, 60.51 ± 2.11%, 58.24 ± 2.03%, and 48.44 ± 1.69%, respectively (Figure 6C). These results indicated that J2 had the best effect on antioxidant capacity compared with other groups.

### 3.8 Antibacterial activity of fermented JA *Jiangshui*

The inhibitory effects of inoculated fermentation and natural fermentation of JA *Jiangshui* on *Staphylococcus aureus*, *Escherichia coli* and *Klebsiella pneumoniae* were determined as shown in Table 4. The inhibition rate of J2 against *Staphylococcus aureus, Escherichia coli* and *Staphylococcus aureus* were the greatest, followed by the J3, J1, and CK. The inhibition zone of J2 against *Staphylococcus aureus, Escherichia coli* and *Klebsiella pneumoniae* were 12.2 ± 0.24 mm, 8.5 ± 0.21 mm and 9.6 ± 0.15 mm, respectively, showing no significant change compared with the J3, but significantly increased compared with J1 and CK. For the three pathogenic bacteria, J2 had the best inhibitory effect on *Staphylococcus aureus*, followed by *Klebsiella pneumoniae* and *Escherichia coli*. Although the mixed bacterial group exhibited a weaker inhibitory effect on pathogenic bacteria compared to kanamycin, it demonstrated stronger antimicrobial activity than the other fermentation methods.

**Table 4:** The inhibitory effect of inoculated fermentation and natural fermentation of JA *Jiangshui* on three pathogenic bacteria (*Staphylococcus aureus*, *Escherichia coli*, *Klebsiella pneumoniae*) was determined.

Pathogenic bacteria	Fermentation method (mm)				Kanamycin
	J1	J2	J3	CK	
*Staphylococcus aureus*	10.3 ± 0.15^b^	12.2 ± 0.24^a^	11.8 ± 0.35^a^	5.5 ± 0.33^c^	24.8 ± 0.28
*Escherichia coli*	6.5 ± 0.13^b^	8.5 ± 0.21^a^	8.2 ± 0.17^a^	4.2 ± 0.24^c^	16.1 ± 0.38
*Klebsiella pneumoniae*	7.8 ± 0.32^b^	9.6 ± 0.15^a^	9.5 ± 0.24^a^	5.2 ± 0.17^c^	18.4 ± 0.35

J1: LAB 815 fermented JA *Jiangshui*; J2: LAB 815 and SC 8(3) mixed fermented JA *Jiangshui*; J3: JA *Jiangshui* fermented with traditional starter culture (*Jiangshui* Yinzi); CK: natural fermentation of JA tuber. All values in the table were mean ± standard deviation (*n* = 3). The mean values of different superscripts in the same column indicated significant differences between strains (*p* < 0.05).

### 3.9 Volatile flavor compounds of fermented JA *Jiangshui*

Volatile compounds are one of the main qualities of *Jiangshui* products, which will affect customer acceptance. In this study, the volatile compounds of pre-fermentation and post-fermentation of JA by the combined inoculation of LAB 815 and SC 8 (3) were identified and quantified. It was found that there was a total of 34 volatile compounds after fermentation, including 10 organic acids, 9 esters, 4 alcohols, 3 ketones, 2 aldehydes, 1 alkene, 1 phenol, etc. After probiotics action, 32 new compounds were produced (Table 5). This study showed that after J2 fermentation, JA produced the most volatile organic acids, accounting for 13.463% of the total mass of *Jiangshui*, followed by alcohols, esters, alkenes, phenols, aldehydes and ketones. The alcohols and esters accounted for 13.414 and 8.344% of the total mass of *Jiangshui*, respectively. The content of phenolic compounds of post-fermentation by J2 was lower than other groups. However, the organic acids, esters, olefins, aldehydes and ketones were significantly higher than other groups (Table 5).

**Table 5:** The content of volatile compounds in inoculated fermentation, natural fermentation JA *Jiangshui* and unfermented JA *Jiangshui* (%).

Retention time (min)	CAS	Compound name	Relative content (%) Fermentation type				
			Without fermentation	J1	J2	J3	CK
Organic acid							
4.677	107-92-6	Butanoic acid	ND	ND	3.301^a^	ND	ND
4.902	16909-11-8	3,5-Dimethoxycinnamic acid	ND	ND	0.477^a^	ND	ND
5.513	503-74-2	Butanoic acid, 3-methyl-	ND	3.039^a^	0.898^c^	1.924^b^	0.191^d^
9.073	142-62-1	Hexanoic acid	ND	ND	0.285^a^	ND	ND
14.559	124-07-2	Octanoic acid	ND	1.195^c^	2.015^a^	0.613^d^	1.297^b^
17.155	112-05-0	Nonanoic acid	ND	ND	0.856^a^	0.271^c^	0.683^b^
19.816	334-48-5	n-Decanoic acid	ND	2.166^b^	2.670^a^	0.428^d^	0.790^c^
24.518	143-07-7	Dodecanoic acid	ND	ND	1.027^a^	ND	0.501^b^
34.946	57-10-3	n-Hexadecanoic acid	ND	1.718^b^	1.341^d^	1.353^c^	2.583^a^
38.046	60-33-3	Linoleic	4.324^a^	ND	0.593^b^	ND	ND
Subtotal	–	–	4.324^e^	8.388^b^	13.463^a^	4.589^d^	6.045^c^
Olefins							
23.221	495-61-4	beta.-Bisabolene	3.745^b^	6.693^a^	3.626^c^	ND	3.190^d^
Subtotal	–	–	3.745^b^	6.693^a^	3.626^c^	ND	3.190^d^
Alcohols							
12.623	60-12-8	Phenylethyl Alcohol	ND	23.283^a^	11.1^b^	5.821^c^	ND
14.852	10482-56-1	L-.alpha.-Terpineol	ND	ND	1.334^a^	ND	0.465^b^
16.806	586-81-2	p-Menth-4 (8)-en-1-ol	ND	ND	0.685^a^	ND	ND
17.892	1653-30-1	2-Undecanol	ND	ND	0.295^a^	ND	ND
Subtotal	–	–	ND	23.383^a^	13.414^b^	5.821^c^	0.465^d^
Aldehydes							
10.593	122-78-1	Benzeneacetaldehyde	ND	ND	0.545^a^	ND	ND
17.154	26254-89-7	2,2-Diethylbutyraldehyde	ND	ND	0.361^a^	ND	ND
Subtotal	–	–	ND	ND	0.906^a^	ND	ND
Esters							
14.983	106-32-1	Octanoic acid, ethyl ester	ND	ND	0.094^a^	ND	ND
16.674	104-45-7	Acetic acid, 2-phenylethyl ester	ND	1.379^b^	1.452^a^	ND	ND
18.482	110-42-9	Decanoic acid, methyl ester	ND	0.141^a^	0.105^b^	ND	ND
20.346	110-38-3	Decanoic acid, ethyl ester	ND	0.561^b^	0.946^a^	ND	ND
21.545	103-48-0	Propanoic acid, 2-methyl-, 2-phen ylethyl ester	ND	ND	4.854^a^	2.700^b^	ND
22.699	140-26-1	Benzylcarbinyl3-methylbutanoat	ND	ND	0.207^a^	ND	ND
25.187	106-33-2	Dodecanoic acid, ethyl ester	ND	0.163^a^	0.156^b^	ND	ND
33.946	112-39-0	Hexadecanoic acid, methyl ester	ND	0.633^a^	0.202^c^	0.394^b^	ND
35.611	628-97-7	Hexadecanoic acid, ethyl ester	ND	0.390^a^	0.328^b^	0.325^c^	0.143^d^
Subtotal	–	–	ND	3.267^c^	8.344^a^	3.419^b^	0.143^d^
Ketones							
10.141	625-33-2	3-Penten-2-one	ND	ND	2.381^a^	ND	ND
17.150	115-67-3	Paramethadione	ND	ND	0.196^a^	ND	ND
21.811	3796-70-1	Geranylaceton	ND	ND	0.197^a^	0.159^b^	0.118^c^
Subtotal	–	–	ND	ND	2.774^a^	0.159^b^	0.118^c^
Phenols							
23.333	128-37-0	Butylated Hydroxytoluene	ND	ND	3.246^a^	ND	ND
Subtotal	–	–	ND	ND	3.246^a^	ND	ND
Others							
10.154	6086-21-1	1-Methyl-1H-1,2,4-triazole	ND	ND	2.086^a^	ND	ND
14.834	636-41-9	1H-Pyrrole, 2-methyl-	ND	ND	0.133^a^	ND	ND
15.534	6308-98-1	Benzeneethanamine, N-(2-phenylethyl)-	ND	ND	3.606^a^	ND	ND
20.333	51-52-5	Propylthiouracil	ND	ND	0.140^a^	ND	ND
Subtotal	–	–	ND	ND	5.955^a^	ND	ND

Without fermentation: Unfermented JA tubers; J1: LAB 815 fermented JA *Jiangshui*; J2: LAB 815 and SC 8(3) mixed fermented JA *Jiangshui*; J3: JA *Jiangshui* fermented with *Jiangshui* Yinzi; CK: natural fermentation of JA tuber. ND: volatile compounds not detected. All values in the table were mean (*n* = 3). ^a–e^Different letters in the same row indicate significant differences (*p* < 0.05).

### 3.10 Amino acid content of fermented JA *Jiangshui*

Glutamic acid, aspartic acid, lysine, and threonine are key amino acids contributing to umami flavor; therefore, their concentrations were specifically measured (Table 6). Our study found that after inoculating fermentation with probiotics, the contents of these four amino acids all increased, but the cysteine was decreased. The glutamic acid content in J1 and J2 increased the most, followed by the J3 and CK compared with the pre-fermentation. Meanwhile, the aspartic acid content in J2 increased the most, approximately 4.3 times that before fermentation, followed by the J3, J1, and CK. For the lysine, the content in J2 increased the most, approximately 8.4 times that before fermentation. The content in CK increased the least and there was no significant difference between J1 and J3. Moreover, for the threonine, the content in J2 increased the most, approximately 5.4 times that before fermentation, followed by the J3, J1, and CK.

**Table 6:** The amino acid content (g/100 mL) of inoculated fermentation, natural fermentation and unfermented JA *Jiangshui*.

Amino acid (mg/L)	Fermentation type				
	Without fermentation	J1	J2	J3	CK
Alanine	ND	0.014^bc^	0.020^a^	0.014^b^	0.0098^c^
Arginine	0.0070^e^	0.0086^d^	0.0090^c^	0.011^b^	0.015^a^
Aspartic acid	0.0051^e^	0.016^c^	0.022^a^	0.018^b^	0.011^d^
Cysteine	0.0013^b^	0.0014^b^	0.00098^c^	0.0017^a^	0.00070^d^
Glutamic acid	0.027^c^	0.044^a^	0.033^a^	0.031^b^	0.028^c^
Glycine	0.0026^d^	0.0078^b^	0.010^a^	0.0077^b^	0.0056^c^
Histidine	0.0017^e^	0.0044^c^	0.0048^a^	0.0039^b^	0.0031^d^
Isoleucine	0.0019^e^	0.0062^c^	0.0091^a^	0.0074^b^	0.0042^d^
Leucine	0.0049^d^	0.011^b^	0.015^a^	0.012^b^	0.0072^c^
Lysine	0.0019^d^	0.013^b^	0.016^a^	0.014^b^	0.0084^c^
Methionine	ND	ND	ND	ND	ND
Phenylalanine	0.0099^b^	0.012^a^	0.013^a^	0.013^a^	0.01^b^
Proline	0.0093^c^	0.014^a^	0.012^b^	0.013^ab^	0.0088^c^
Serine	0.0036^e^	0.0091^c^	0.011^a^	0.0097^b^	0.0049^d^
Threonine	0.0026^e^	0.01^c^	0.014^a^	0.011^b^	0.0058^d^
Tyrosine	ND	0.005^c^	0.0064^a^	0.0054^b^	ND
Valine	0.0034^d^	0.01^b^	0.013^a^	0.011^b^	0.0065^c^

Without fermentation: Unfermented JA tubers; J1: LAB 815 fermented JA *Jiangshui*; J2: LAB 815 and SC 8(3) mixed fermented JA *Jiangshui*; J3: JA *Jiangshui* fermented with *Jiangshui* Yinzi; CK: natural fermentation of JA tuber. ND: volatile compounds not detected. All values in the table were mean (*n* = 3). ^a–e^Different letters in the same row indicate significant differences (*p* < 0.05).

Before and after the fermentation of JA, no methionine was produced. However, tyrosine was found in J1, J3, and J2, but not in CK. The above research results indicated that the content of umami amino acids was the highest in J2, followed by the J3, J1, and CK.

### 3.11 PH and cell growth of JA *Jiangshui*

**Figure 7:**
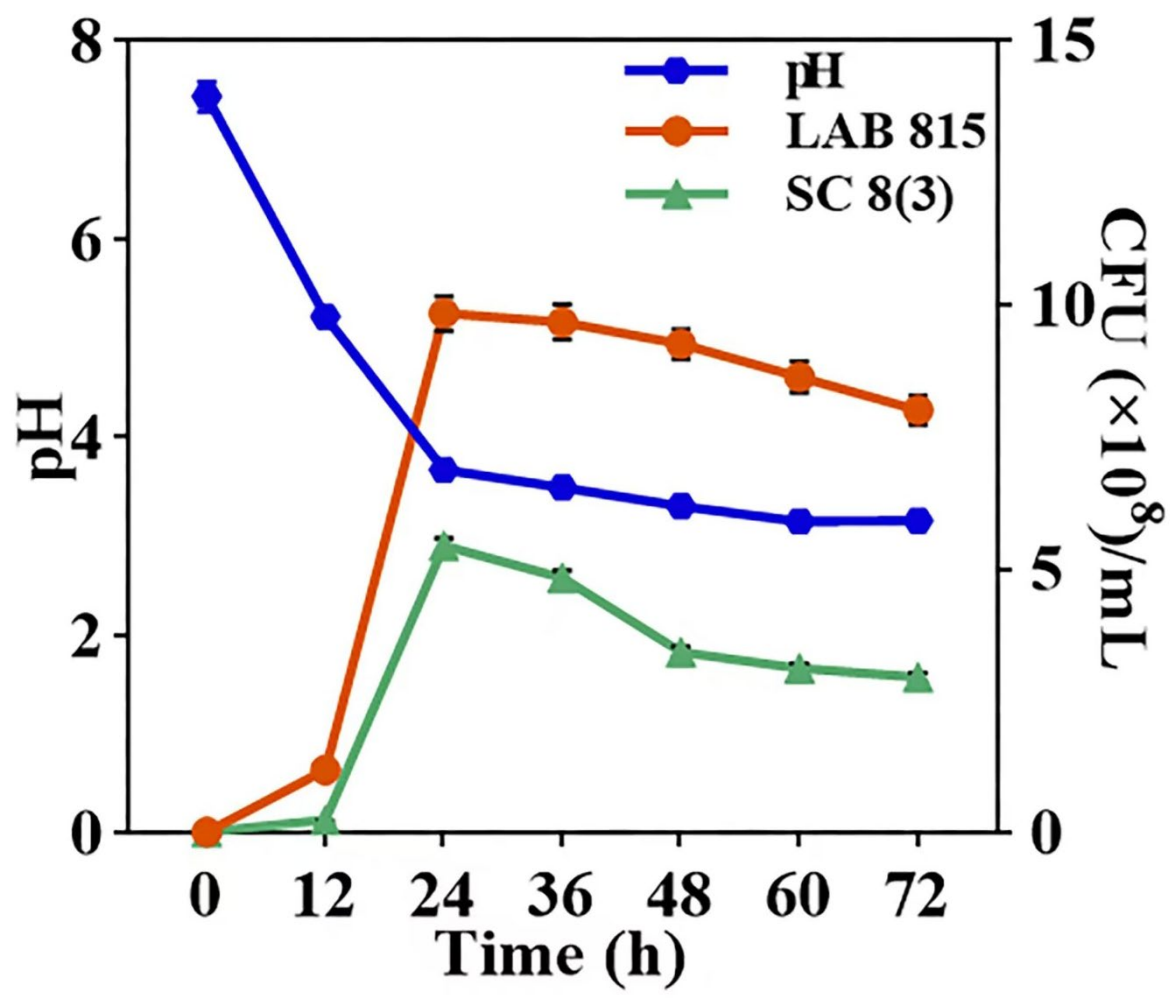
The growth curves of LAB 815 and SC 8(3) as well as the pH value were determined during the fermentation of JA *jiangshui*. Note: All values in the figure were mean ± standard deviation (*n* = 3).

The growth curves and pH values of LAB 815 and SC 8 (3) during JA *jiangshui* fermentation were determined (Figure 7). Both strains grew slowly within the first 12 h, then increased rapidly and reached their maximum cell densities at 24 h: 9.84 × 10^8^ CFU/mL for LAB 815 and 5.44 × 10^8^ CFU/mL for SC 8(3). From 24 to 72 h, cell densities declined slightly and stabilized, indicating that both strains entered a stationary phase after 12 h of rapid growth. During the same period, the pH of the fermentation broth dropped sharply from 7.44 to 3.16 and remained stable at approximately 3.16 after 60 h.

## 4 Discussion

JA tuber is rich in inulin, a type of dietary fiber that functions as a prebiotic, promoting gut health by stimulating the growth of beneficial bacteria in the digestive tract. Several studies have shown that JA inulin can effectively reduce blood glucose and reduce complications (Sawicka et al., 2020; Li et al., 2022). At the same time, inulin can generate short-chain fatty acids under the action of intestinal microorganisms, improve the intestinal barrier function, promote uric acid excretion, and have a certain therapeutic effect on hyperuricemia (Fan et al., 2025). These beneficial effects highlight inulin’s potential as a functional ingredient in dietary strategies for diabetes and hyperuricemia management. Probiotics, such as LAB, have been shown to alleviate diabetes and hyperuricemia through a variety of mechanisms. A study by Wang et al. (2020) showed that *Bifidobacterium adolescentis*, *B. bifidum* and *Lactobacillus rhamnosus* can reduce fasting and postprandial blood glucose levels, improve glucose tolerance, and prevent pancreatic damage in type 2 diabetic mice induced by high-fat diet and streptozotocin. Previous studies have demonstrated that *Lactobacillus reuteri* (TSR 332) and *L. fermentum* (TSF 331) can help treat hyperuricemia by utilizing purines and thereby reducing uric acid (UA) synthesis (Kuo et al., 2021). In addition, other Lactobacillus strains, such as *Lactobacillus brevis* (DM 9218) and *L. gasseri* (PA-3), have been shown to effectively manage hyperuricemia by degrading intermediates of purine metabolism (Li et al., 2014; Yamada et al., 2017). Therefore, the present study hypothesized that probiotics combined with JA tubers have a better therapeutic effect on diabetes and hyperuricemia than probiotics or JA tubers. In this study, LAB and yeast were used for the first time to ferment JA tubers as JA *Jiangshui*, and its alleviating effect on diabetes and hyperuricemia was evaluated. Our study stated that the *Jiangshui*-originated LAB we screened has a significant inhibitory effect on α-glucosidase and α-amylase (Table 2), aligning with the findings of Huligere et al. (2023) who reported that *Lactobacillus paracasei* RL-CS, RL-CE and RL-IC inhibited α-amylase (17.25–55.42%) and α-glucosidase (15.08–59.55%). The strain LAB 815 that we screened showed the highest inhibitory effect on α-glucosidase and α-amylase, the inhibition rates could reach 42.50 and 40.11%, respectively, which were 52.69 and 51.31% of acarbose (1 mg/mL) (Table 2). And it was identified as *Lactobacilus paracasei* by the 16S rRNA gene sequence (Figure 2A).

To screen out the most suitable highly hypoglycemic LAB for fermenting JA *Jiangshui*, LAB 815,818 and 833 were co-fermented with JA to determine their hypoglycemic ability *in vitro*, respectively. It was found that the hypoglycemic ability of JA *Jiangshui* fermented with LAB 815 and 818 was significantly higher than that of JA juice, but the LAB 833 was significantly lower than that of JA juice (Figure 1). It was found that LAB 815 fermented JA *Jiangshui* had the highest hypoglycemic ability, and had the highest inhibition rate on α-glucosidase and α-amylase activity, which were 51.23 and 42.50%, respectively (Figure 1). Several studies have suggested that both lactic acid bacteria (LAB) and Jerusalem artichoke (JA) possess notable hypoglycemic effects (Kim et al., 2013; Wang et al., 2020). Supporting this, Wan et al. (2019) demonstrated that probiotic-fermented carrot pulp was more effective in reducing blood glucose levels compared to unfermented carrot pulp. Qi et al. (2024) proved that after probiotic fermentation can improve the *in vitro* antioxidant and hypoglycemic capacity of wolfberry juice, thus improving the nutritional value and function of wolfberry juice to a certain extent. Similar findings were observed in the current study, and this improvement may be attributed to the metabolic activity of the LAB strains during fermentation. In contrast, LAB strain 833 appeared less effective, possibly due to its poor adaptability to the high acidity of *Jiangshui*, resulting in a reduced hypoglycemic effect.

As a kind of local food in Northwest China, the flavor of *Jiangshui* is very important. Microbial diversity during food fermentation could enriches the flavor of food (Terefe and Augustin, 2020). The dominant strains in the *Jiangshui* are LAB and yeast (Hu et al., 2021). Moreover, LAB not only produce lactic acid during fermentation, giving food sourness, but also produce a variety of vitamins and short-chain fatty acids, which are beneficial to human intestinal health (Farias et al., 2019). Yeasts also play a central role in food fermentation, the fermentation of yeast not only affects the flavor of food, but also improves the texture and taste of food (Ferrocino et al., 2022). Sun et al. (2021) reported that co-fermenting the kiwifruit juice with selected *W. anomalus* strain and *Saccharomyces cerevisiae* not only enriched the types and concentrations of volatiles of kiwifruit wine compared with pure culture fermentation but also had better sensory characteristics. We also obtained the same result, the co-fermentation of JA *Jiangshui* with aroma-producing yeast strain 8(3) and LAB 815 significantly enhances total acid and ester production, improving flavor profiles compared to fermentation with LAB 815 alone (Figure 3).

Bile salt tolerance is a key characteristic enabling lactic acid bacteria (LAB) to survive in the harsh conditions of the small intestine. Probiotics must be able to survive in acidic (pH 1.5–3) stomachs and intestines containing 0.03–3% w/v bile salts (Cai et al., 2019). Previous studies have demonstrated that various microorganisms can withstand acidic environments and bile salts. For instance, Khaled et al. (2018) reported that *Bacillus velezensis* R7-1003 was resistant to low pH (2) and a high bile salt concentration (0.3%), while Wang et al. (2021) found that *Lactobacillus fermentum* 21,828 exhibited good tolerance to pH 2.5 and bile salts. Similarly, Alkalbani et al. (2022) showed that yeasts isolated from both fermented dairy and non-dairy products could survive *in vitro* digestion conditions and had some level of bile salt resistance. In the present study, LAB 815 demonstrated strong acid and bile salt tolerance, with survival rates of 84.15% in acidic medium (pH 2) and 78.24% in 0.4% bile salt after 3 h (Table 3). Likewise, the yeast strain SC 8(3) showed survival rates of 80.21% in acidic conditions and 76.25% in 0.3% bile salt medium after 3 h, which is comparable to findings by Koh et al. (2018), who reported that *Lactobacillus mali* K8 had a survival rate of around 80% under similar conditions.

The adhesion characteristics of probiotics are an important factor in determining their probiotic properties. Animal and clinical studies have shown that weakly adhered LAB are continuously excreted through feces (Henriksson and Conway, 1996). Firm adhesion to the intestinal lining is essential for probiotics to exert their beneficial effects (Moal et al., 2002). This adhesive ability is closely linked to cell surface hydrophobicity and self-aggregation, both of which positively correlate with probiotic adherence (Kang et al., 2017). In the present study, LAB 815 exhibited a hydrophobicity of 37.03% and a self-aggregation rate of 27.24%, while SC 8(3) showed hydrophobicity and self-aggregation values of 36.85 and 20.88%, respectively (Table 3). These hydrophobicity levels are notably higher than those reported for *Leuconostoc mesenteroides* MKSR (6.46%) (Lee and Kim, 2019) and *L. enteroides* KACC 12412 (30.30%) (Xu et al., 2009). However, the self-aggregation of LAB 815 and SC 8(3) was slightly lower than that of previously reported *L. brevis* KU 15006 (33.74%) (Son et al., 2017) and *L. enterovirus* NRRLB-1149 (55.20%) (Shukla et al., 2014). These results indicate that both LAB 815 and SC 8(3) had good intestinal adhesion ability which may enhance their probiotic effectiveness.

Many probiotics have successfully prevented or treated various metabolic diseases, including diabetes and hyperuricemia. Probiotics such as *Lactobacillus paracasei*, *Lactobacillus lactobacilli* (Wang and Li, 2022), *Saccharomyces cerevisiae* (Wu C. H. et al., 2021), *Saccharomyces boulardii* (Cunha et al., 2020) have a certain effect on alleviating diabetes. *Lactobacillus plantarum*, *L. reuteri*, *L. fermentum*, *L. brevis*, *L. garvieae*, and *Saccharomyces cerevisiae* are probiotics that have a certain mitigation effect on hyperuricemia (Cunha et al., 2020). Previous studies have shown that α-glucosidase, α-amylase inhibition rate and glucose dialysis delay index are the key indicators of *in vitro* hypoglycemic ability (Huligere et al., 2023; Chen et al., 2021). Xanthine oxidase (XO) is a key enzyme in the synthesis of uric acid, and XO inhibitors can reduce the production of uric acid (Chen et al., 2017). Qi et al. (2024) reported that after probiotic fermentation of wolfberry juice, the hypoglycemic ability of wolfberry juice was improved, and the inhibition rates of α-glucosidase and α-amylase reached 52.30 and 51.79%, respectively. Pan et al. (2025) reported that the glucose dialysis retardation index of loquat juice fermented by *L. brevis* was the highest after dialysis for 30 min and 60 min, which was 16.49 and 9.76%, respectively. Xu et al. (2021) showed that the xanthine oxidase inhibitory activity of yeast-fermented coix seed was significantly higher than that of unfermented coix seed, reaching about 70%. In this study, compared with JA juice, the inhibition rate of α-glucosidase, α -amylase and xanthine oxidase, and the glucose dialysis delay index of JA *Jiangshui* formed by the combined fermentation of JA with LAB 815 and SC 8(3) were significantly increased (Figure 4). The inhibition rates of α-glucosidase, α-amylase, and xanthine oxidase were 53.26, 61.79, and 83.46%, respectively, while the glucose dialysis retardation index reached 55.67%. These findings are consistent with those reported in previous studies, indicating strong enzyme inhibitory activity and glucose absorption retardation potential (Xu et al., 2021; Qi et al., 2024; Pan et al., 2025). These results indicate that the combined fermentation of JA *Jiangshui* with LAB 815 and SC 8(3) enhanced its *in vitro* ability to lower blood glucose and uric acid levels. Although its hypoglycemic effect was not as potent as that of acarbose, the fermented product still shows promising potential as a natural agent for managing blood glucose and uric acid. To function as a probiotic in the human body, it must be able to tolerate acidic environments in the stomach and intestines at pH 2 and 8, respectively, as well as tolerance to bile acid concentrations and antagonism to pathogenic organisms (Fooks et al., 1999). The gastrointestinal fluid simulation experiments showed that the ability to lower blood glucose and uric acid *in vitro* of JA *Jiangshui* fermented by different fermentation methods decreased to a certain extent during the whole incubation period. Among all fermentation treatment, J2 showed the strongest *in vitro* inhibitory effects on α-glucosidase, α-amylase, and xanthine oxidase, and maintained the highest stability in lowering blood glucose and uric acid after gastroenteric fluid treatment (Figure 5). These results suggest that LAB 815 and SC 8(3) can work synergistically and effectively resist the highly acidic environment within the gastrointestinal tract.

Previous studies reported that LAB fermentation had a positive impact on the *in vitro* antioxidant activity and antibacterial activity of fermented foods (Rocchetti et al., 2019). As such Chen et al. (2020) reported that higher antioxidant potential in fermented BS compared to unfermented BS. Further, Hashemi et al. (2021) reported that fermentation of peach juice by lactic acid bacteria effectively inhibited the growth of Salmonella freiburg. Previously, it has stated that organic acids (lactic acid, acetic acid, etc.) and flavonoids in *Jiangshui* effectively inhibit the growth of some spoilage bacteria (Zhang et al., 2024). So, the influences of JA *Jiangshui* fermented with LAB 815 and SC 8(3) on antioxidant activities and antibacterial activities were further investigated. The results showed that JA *Jiangshui* co-fermented with LAB 815 and SC 8(3) exhibited the strongest scavenging effects on DPPH, hydroxyl, and superoxide anion radicals (Figure 6), and had a good inhibitory effect on *Staphylococcus aureus*, *Escherichia coli* and Klebsiella as compared with the CK (Table 4). Although J2’s inhibitory effect on pathogenic bacteria was less than that of kanamycin, it still demonstrated notable antimicrobial activity. These results indicate that JA *Jiangshui* co-fermented by LAB 815 and SC 8(3) had significant antioxidant activity and antibacterial activity. The bacteriostatic effect of fermented JA *Jiangshui* may come from two aspects, on the one hand, flavonoids have a certain bacteriostatic effect (Chagas et al., 2022). On the other hand, carbon dioxide, hydrogen peroxide, acidic substances, peptides and proteins produced by lactic acid bacteria have a wide range of inhibitory effects (Liu et al., 2022). As the determination of volatile substances (Table 5), the content of organic acids and ketones in JA *Jiangshui* significantly increased by LAB 815 and SC 8(3) fermentation, so had a significant inhibitory effect on the growth of some spoilage bacteria.

Besides this, volatile flavor compounds and amino acids are important indicators of the quality and texture of pickled vegetables and can help improve their sensory properties (Liang et al., 2020). Studies shown that organic acid has an important influence on the flavor and stability as the most important substance in *Jiangshui*; Butyric acid, which has a strong cheese flavor and sour taste, can enhance the sourness and complexity (Hu et al., 2025); Aldehydes and ketones mainly provide fragrance and aromatic characteristics; Hydrocarbons provide lemon aroma and resin aroma; Alcohols mainly provide plant aroma and ester aroma; esters mainly provide fruit aroma and wine aroma (Xu et al., 2024). Juan Gilberto et al. (2022) also showed that mannitol produced by LAB can give pickles a sweet and refreshing taste, while myrcene, limonene, linalool, geraniol, methyl butyrate and other substances have been identified as flavor contributors to pickles. Our study showed that 28 new compounds were generated in JA *Jiangshui* by co-fermentation of LAB 815 and SC 8(3) (Table 5). The volatile compounds, in decreasing order of abundance, were organic acids, alcohols, esters, olefins, phenols, aldehydes and ketones. The amino acids that affect the umami flavor are glutamic acid and aspartic acid (Park et al., 2024). Our study found that probiotic fermentation increased the levels of both amino acids, with the highest increase observed in JA *Jiangshui* co-fermented with LAB 815 and SC 8(3), consistent with previous findings (Park et al., 2024). These results indicated that JA *Jiangshui* fermented with the co-culture of LAB 815 and SC 8(3) has a better flavor. Through determining the growth curves of LAB 815 and SC 8(3) as well as the pH value during the fermentation of JA *jiangshui*, we found that both strains grew well (Figure 7).

## 5 Conclusion

In this study, LAB 815 with high hypoglycemic properties was isolated from Northwest *Jiangshui* could significantly inhibit α-glucosidase and α-amylase. Through 16S rRNA gene amplification and sequencing, the strain was identified as *Lactobacillus paracasei* with homology similarity of 100%. Meanwhile, the most suitable aroma-producing yeast SC 8(3) was selected for the co-fermentation of JA *Jiangshui* with LAB 815 by the total ester and total acid contents. Both LAB 815 and SC 8(3) had good acidity resistance and bile salt tolerance and could adhere to the intestinal epithelium. JA *Jiangshui* formed by the combined fermentation of JA with LAB 815 and SC 8(3) had a significant inhibitory effect on α-amylase, α-glucosidase, glucose dialysis delay index, antioxidant activity and antibacterial activity, indicated that the joint fermentation of JA with LAB 815 and SC 8(3) significantly improves the biological activity function of JA itself. The gastrointestinal fluid simulation experiments showed that JA *Jiangshui* fermented with LAB 815 and SC 8(3) exhibited the least decrease in the inhibition rate of diabetes and hyperuricemia indicator enzymes, indicating that its ability to reduce blood glucose and uric acid *in vitro* was alleviated after exposure to gastrointestinal fluid,. Those results shown that the cooperation between LAB 815 and SC 8(3) maybe greatly mitigates the adverse effects of the highly acidic gastrointestinal environment. The significant increase in organic acids, ketones, volatile compounds, and amino acids in JA *Jiangshui* fermented with LAB 815 and SC 8(3) suggests enhanced flavor and stronger inhibitory effects against spoilage bacteria. The present study lays a foundation for the development of functional fermented foods.

## Data Availability

The datasets presented in this study can be found in online repositories. The names of the repository/repositories and accession number(s) can be found in the article/supplementary material.
